# Assessment of cancers’ diagnostic stage in a Deaf community - survey about 4363 Deaf patients recorded in French units

**DOI:** 10.1186/s12885-017-3972-3

**Published:** 2018-01-23

**Authors:** Vladimir Druel, Hélène Hayet, Laetitia Esman, Marie Clavel, Marie-Eve Rougé Bugat

**Affiliations:** 1 University Department of General Practice, Toulouse-Rangueil Faculty of Medicine, 133 route de Narbonne, 31400 Toulouse, France; 2 Oncology united, Auch Hospital, Allée Marie Clarac, 32008 Auch, France; 3 General practitioner in the medical board of Auch, ‘Pion’, 32190 Lannepax, France; 4 Deaf Care Unit, Teaching Hospital of Toulouse-Purpan, Place du Dr Joseph Baylac, 31300 Toulouse, France; 5 Deaf Care Unit, Teaching Hospital of Grenoble, Avenue Maquis du Grésivaudan, 38700 La Tronche, France; 6 Inserm U1027, Faculty of Medicine, 37 allées Jules Guesde, 31073 Toulouse, France; 7 DESC Oncology, 133 route de Narbonne, 31000 Toulouse, France

**Keywords:** Cancer, Deaf, General practice, Communication, Health inequalities, Delayed diagnosis

## Abstract

**Background:**

Deaf people represent 0.1% of the French population and their access to public health campaigns is limited due to their frequent illiteracy and the infrequent use of sign language in campaigns. There is also a lack of general health knowledge in spite of the existence of French Deaf Care Units (UASS). The aim of this study is to assess the average diagnostic stage of cancer in the Deaf Community and discuss deafness as a contributing factor.

**Methods:**

Four thousand three hundred sixty-three Deaf patients recorded in five UASS, 80 diagnosed between 2005/01/01 and 2014/12/31 were selected from medical records and/or ICD-10 coding. Data regarding cancers were extracted, grouped by stage and compared to literature. Statistical significance was tested with Fisher’s Exact Test.

**Results:**

Eighty patients were selected. Most cancers were diagnosed at advanced stages: of 11 prostate cancers, 46% were locally advanced and 18% were metastatic. (In the general population, this was respectively 3% and 10.4% (*p* < 0.01)). Of six colorectal cancers, 67% were diagnosed at stage III and 33% at stage IV. (Respectively 20.6% and 26.6% (*p* = 0.03) in the general population). In contrast, of the 15 breast cancers, 93% were diagnosed at stages T1-T3 that was earlier than in the general population (*p* = 0.43).

**Conclusion:**

In this study, we observed a delay cancer diagnosis among Deaf people. Complicated and/or non-systematic screening procedures for cancers would be involved. Which is most likely the result of many factors (communication, medical knowledge). Increasing UASS coverage and health information campaigns in sign language could assist in earlier cancer diagnosis.

## Background

In 2010, the World Health Organization (WHO) estimated that the world population suffering from moderate to severe hearing loss was 360 million, including 32 million children [1] who tended to use sign language more often. In France, an estimated 5.5 million people were suffering from moderate to severe hearing impairment in 2008 (about 9.2% of the general population). Among those who suffered from deafness before the age of six years, only 1% (about 51,000 persons) utilized French Sign Language (FSL) instead of French as their main language [2]. These people generally have shared the same culture and have been referred to as Deaf [3]. For them, situations of social and cultural exclusion have been common. Indeed, until 1991, the ban of FSL in education led to a severe communication handicap [4]. In 2013, 60 to 80% of this population was considered illiterate or had imperfect mastery of written language [5] and socioeconomic status deprivation is a significant independent determinant for stage of diagnosis and survival [6–8]. This limits their access to information, including public health prevention campaigns [9–11]. For the most part, the Deaf have had very little general health knowledge [12]. Founded in 1995, the French Deaf Care Units, “Unités d’Accueil et de Soins pour les Sourds” (UASS) have been providing healthcare utilizing FSL [13]. The UASS makes possible to carry out a general practice’s consultation (treating and sometimes addressing to an organ specialist or oncologist). Since its foundation, a total of 19 UASS have opened throughout France.

This limited access to healthcare could have far-reaching consequences for the Deaf such as delayed cancer diagnosis, with cancer being the leading cause of death in France [14]. As a matter of fact, the worldwide cancer diagnosed population has increased to 35.2 million in 2012 [15] from 28 million in 2008 [16]. In France, social disparities have been observed in access to health care [17]. The government has created a National Cancer Plan to study those disparities and improve access to health care [18]. But no incidence or prevalence data has been published to date, regarding the Deaf population [19].

This study makes an assessment of cancers in deaf people and their diagnostic stage followed medically by five UASS and subsequently compared to the general population.

## Methods

### Study design and population

A descriptive, retrospective, multicentric study was carried out using data, collected during a 10 year period, from five French UASS connected to the University Hospitals of Grenoble, Marseille, Toulouse, Poitiers and Paris. Patients’ medical records from Paris and Poitiers were preselected with International Classification of Diseases codes (ICD-10) for cancer, complication of cancer or pre-cancerous lesion [20], while data from Grenoble, Marseille and Toulouse were extracted manually in addition to the utilization of ICD-10 codes. After preselection, the retained records where reviewed manually.

This selection process involved cases of cancer diagnosed between 2005/01/01 and 2014/12/31 in Deaf patients who received medical care at a UASS. There are no socio-demographic criteria of exclusion for the admission in the UASS; it’s the only medical unit in France for the Deafs people. Patients without deafness were excluded (even if followed by UASS) as well as patients suffering from pre-cancerous lesions or cancer recurrence. Assessment criteria included demographic elements (sex and age at diagnosis) and cancer characteristics (organ targeted, diagnostic stage, pathology results).

### Procedures

This study used the diagnostic stage of cancer defined by multidisciplinary meetings, or, failing that, the stage reported in the summary of the pathologist. Data were grouped in stages for each family of cancer and compared to the most recent data from the general French population. For example, prostate cancer control data were derived from a French population study dating from 2001 [21]. Regarding breast cancer, reference data were extracted from 2000 to 2010 three French area [22]. For all other cancers the reference study [23] was based on data from PETRI (a cohort analysis concerning the mortality of cancer patients in France between 1994 and 1999 [24]) and the American SEER program taken from 1999 to 2005 (cancer incidence in the USA) [25].

The 3 most frequent cancers (breast, prostate and colo-rectal) were compared to the literature on the general population. It is described in detail in the “Results” section, and was realized to allow better data analysis and appreciation of the results.

### Statistical analysis

A descriptive analysis of the patients’ characteristics was conducted, As well as a descriptive analysis of their cancer and his stage of diagnosis. Categorical variables were expressed as percentage frequencies. The significance of the data was confirmed by Fisher’s Exact Test (FET) using the programming language R (version: 3.1.3) with *p* < 0.05.

## Results

### Characteristics of the population

**Fig. 1:**
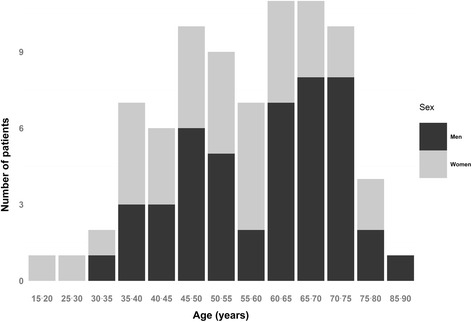
Distribution of the Deaf patients according to age

The population represented in this study included 4363 people followed in five UASS. Among them, 85 were diagnosed with cancer between 2005/01/01 and 2014/12/31. One patient was excluded because the origin of his cancer was not identified (discovered at an overly advanced stage). Four patients were excluded because of their age, less than 18 years old (possibly related with oncological pathology of childhood or related to a genetic disease possibly related itself to the cancer appearance). Of the 80 patients remaining, 34 were women and 46 were men. The average age of the sample was 54.55 years, ranging between 19 and 88 years of age (Fig. 1).

The cancer distribution included 9 onco-hematologic cases and 71 solid cancers with 10 cases involving metastasis (Table 1). Overall the distribution was different between Deaf and referent populations [21–23] (Table 2). The most represented cancers were breast cancer (with 0.34% of the 4363 screened deaf people - 15 patients), prostate cancer (0.25% - 11 patients), and with the same rates, colo-rectal cancer and basal-cell carcinoma (0.18% - 8 patients).

**Table 1:** Assessment of cancers’ diagnostic stage in a Deaf Community in this study

Cancer (*n* (%))	Diagnostic stages					
	Stage 0	Stage I	Stage II	Stage III	Stage IV	NI^a^
Colorectal (*n* = 8)	2	–	–	4	2	–
Melanoma (*n* = 3)	–	–		1	2	–
Stomach (*n* = 2)	–	–	1	–	1	–
Hodgkin’s lymphoma (*n* = 3)	–	–	–	2	–	1
Osteosarcoma (*n* = 1)	–	–	–	1	–	–
Pancreatic (*n* = 1)	–	–	–	–	1	–
Lung (*n* = 3)	–	–	1	–	2	–
Kidney (*n* = 2)	–	1	–	1	–	–
Thyroid (*n* = 3)	–	2	1	–	–	–
	T1N0M0	T2/T3N0M0	T1/T3 N + M0	T4 N + M0	M+	NI
Breast (*n* = 15)	3	5	6	–	–	1
	T1N0M0	T2N0M0	T3/T4N0M0	N + M+	NI	
Prostate (*n* = 11)	1	1	5	2	2	
	Localized	Regional	Metastatic			
Oropharyngeal (*n* = 1)	–	1	–			
Basal cell carcinoma (*n* = 8)	8	–	–			
Squamous cell carcinoma (*n* = 3)	3	–	–			
Brain glioma (*n* = 1)	1	–	–			
Cervical (*n* = 1)	1	–	–			
NHL^b^ (*n* = 4)	1	2	1			
Ovarian (*n* = 1)	–	–	1			
Testicular (*n* = 3)	3	–	–			
Uterine (*n* = 3)	2	–	1			
	Remarks					
Leukemia (*n* = 2)	1 AML^c^,					
	1 B-CLL^d^					
Thymoma (*n* = 1)	1					

*ND* Not Definited

^a^: stage indefinite. ^b^: Non-Hodgkin’s lymphoma. ^c^: acute myeloid leukemia

^d^: chronic lymphocytic leukemia of B cells

**Table 2:** Comparison of this assessment to baseline studies: only presenting cancers cases (except testicle)

Type of cancer (number of Deaf cases)	Stage	% observed in the sample studied (nomber)	% of the reference population	Baseline studies
Colo-rectal (*n* = 6)	I	–	25.7%	PETRI (Fr) [24] (37,413 patients)
	II	–	21.3%	
	III	67% (4)	20.6%	
	IV	33% (2)	26.5%	
	ND^a^	–	5.9%	
Melanoma (*n* = 3)	I and II	–	86.2%	PETRI (Fr) [24] (7401 patients)
	III	33% (1)	3.4%	
	IV	67% (2)	6.4%	
	ND^a^	–	4%	
Hodgkin’s lymphoma (*n* = 3)	I	–	81.7%	PETRI (Fr) [24] (1544 patients)
	II	–	8.7%	
	III	67% (2)	3.8%	
	IV	–	3.8%	
	ND^a^	33% (1)	2.0%	
Non-Hodgkin’s lymphoma (*n* = 4)	Localized	25% (1)	30%	SEER (USA) [25] (10,224 patients)
	Regional	50% (2)	15%	
	Metastatic	25% (1)	47%	
	ND^a^	–	9%	
Lung (*n* = 3)	I	–	15.5%	PETRI (Fr) [24] (31,557 patients)
	II	33% (1)	13.6%	
	III	–	20%	
	IV	67% (2)	39.4%	
	ND^a^	–	11.5%	
Prostate (*n* = 11)	T1N0M0	9% (1)	27.4%	« *Cancer de la prostate en France en 2001* » [21] (2181 patients)
	T2N0M0	9% (1)	59.2%	
	T3/T4N0M0	46% (5)	3.0%	
	N + M+	18% (2)	10.4%	
	ND^a^	18% (2)	–	
Breast (*n* = 15)	T1	33% (5)	66%	*Breast cancer incidence: Decreasing trend in large tumours in women aged 50–74* [22] (28,092 patients)
	T2+	60% (9)	34%	
	ND^a^	7% (1)	–	
	N0	53% (8)	63%	
	N+	40% (6)	33%	
	ND^a^	7% (1)	4%	
	M0	93% (14)	90.5%	
	M+	1 (7%)	4.5%	
	ND^a^	–	5%	
Thyroid (*n* = 3)	I	67% (2)	67.9%	PETRI (Fr) [24] (6672 patients)
	II	33% (1)	6.4%	
	III	–	9.2%	
	IV	–	9.2%	
	ND^a^	–	7.3%	
Uterine (*n* = 3)	Localized	67% (2)	69%	SEER (USA) [25] (5774 patients)
	Regional	–	19%	
	Metastatic	33% (1)	8%	
	ND^a^	–	4%	

^a^ND: Not Definited

### Cancer’s stage in the deaf population compared to general population

Table 2 showed the cancers with a similar distribution as in the general population and cancers who were diagnosed at later stages such as prostate cancer and colorectal cancer or melanoma.

From the 3 types of cancer most frequent in our study, the first cancer was breast cancer with 15 cases, half of them at a local stage (N0). This is not significantly different from the general population (*p* = 0,58) [22]. We found 6,7% of metastatic breast cancer in the deaf population and 4,5% in the general population (*p* = 0,52). This is in favour of a similar repartition of the different stages of diagnosis, even if the very early stage T1 is slightly more present in the general population (Table 3).

**Table 3:** Analysis of the differences of this assessment to baseline studies: only presenting colo-rectal, prostate and breast cancer (more 6 cases)

Type of cancer (number of Deaf cases)	Stage	Number observed in the sample studied (nomber)	% of the reference population	*p-values*. fisher’s exact test	Baseline studies
Colo-rectal (*n* = 6)	I/II	–	47% (17584)	0.03	PETRI (Fr) [24] (37,413 patients)
	III/IV	100% (6)	47% (17622)		
	ND^a^	–	6% (2207)		
Prostate (*n* = 11)	T1–2/N0	18% (2)	87% (1889)	0.04	« *Cancer de la prostate en France en 2001* » [21] (2181 patients)
	T3+ or N+ or M+	64% (7)	13% (292)		
	ND^a^	18% (2)	–		
Breast (*n* = 15)	T1	33% (5)	66% (18404)	0.02	« *Breast cancer incidence: Decreasing trend in large tumours in women aged 50–74* » [22] (28,092 patients)
	T2+	60% (9)	34% (9688)		
	ND^a^	7% (1)	–		
	N0	53% (8)	63% (17696)	0.58	
	N+	40% (6)	33% (9205)		
	ND^a^	7% (1)	4% (1191)		
	M0	93% (14)	90.5% (25434)	1	
	M+	1 (6.7%)	4.5% (1268)		
	ND^a^	–	5% (1390)		

^a^ND: Not Definited

On the contrary, of 11 prostate cancers diagnosed in the studied sample, 64% were found at an advanced local or metastatic stage. This level of prostate cancer advancement was particularly high since, in the literature data of the general population, only 13% were diagnosed at this stage [21] (*p* = 0.04).

The six colorectal cancers found in the Deaf sample were primarily far advanced at diagnosis: all are diagnose at stage III and IV when in the general population, 47% were diagnosed at stage I and II, and the same amount at stage III and IV [24] (*p* = 0.03).

## Discussion

Our work reviewed 4263 patients’ in five UASS (Grenoble, Marseille, Paris, Poitiers and Toulouse) in, France and 80 Deafs’ cancer patients. Three groups of cancers emerge from the study, depending on early, similar, or late diagnosis, compared to the general population.

### Comparison with existing literature

Of all breast cancers in the study, one was metastatic or T4 (TNM) stage. The diagnosis appeared similar in the Deaf population than in the general population (*p* = 0. 58; *p* = 0.52). However, these findings could be linked to the Orsi study [10] which showed the same amount of mammography performed for hearing and Deaf Americans [26]. This could be explained by the simplicity of this screening’s availability (a simple prescription, a third party performs the test). Furthermore, French Social Security systematically offers this screening. Increasing mammographic screening coverage reduce late-stage cancer at diagnosis and to reduce socioeconomic disparities [27, 28]. It allows the Deaf patient to have a visual understanding of mammography: the FSL sign of mammography is now used in common language so the patient can use it to spread the information within the community.

On the contrary, colo-rectal cancer (*p* = 0.03), prostate cancer (*p* = 0.04) and melanoma were diagnosed at a more advanced stage in the Deaf population than in the general population. A delayed diagnosis for the Deaf was observed in these samples. The colo-rectal cancer’s screening test used until 2015 (“Hemoccult II ®”) is difficult to explain [29] and even more when there are language barriers or when patients are partially illiterate. This test has to be performed by the patient and if the objective or performance of the test is not understood, completion of the test would be considerably reduced compared to the general population.

For prostate cancer, the rectal examination must be explained prior to being performed. According to Orsi’s study, there was more frequent performance of the rectal examination in Deaf people than in the general population observed in the USA [10]. Prostate cancer testing, which is the combination of rectal examination and measure of the prostate specific antigen (PSA), is not a mass screening but is chosen case by case. The decision to undergo prostate cancer screening is made between physician and patient rather than by societal education or medical protocol. This might explain the delayed diagnosis in the Deaf population, as the communication issue remains important. Moreover, we observed that the frequency of PSA testing can be increased by the concern of patients, consequently at their knowledge of the cancer, particularly before the age of 60 [30], and with a higher socioeconomic status [31]. This limited access to the individual screening could have limited the increased over diagnosis (and treatments) performed on the general population for prostate cancer. Screening prostate cancer didn’t prove a significative decrease of mortality [32] but the decrease of quality of life [33]. For melanoma, it appears that the instructions for auto-monitoring and prevention involve the same issue of communication.

### Strengths and limitations

There is a recruitment issue: only patients from UASS have been included. Although this recruitment allows the best reflection of a Deaf population without a national register of Deaf people, these units mainly select Deaf people with complex medical or social issues. Another selection bias is that the medical records have been manually selected or selected according to ICD-10 coding. In some cities, the computer systems cannot include all oncology units, which are spread throughout many different hospitals.

In addition, the sample size was small, thus, creating limitations to the results’ significance. This is, however, the first study about this topic and the results are relevant for a number of cancers. The comparison to the data of the literature is only indicative.

Second of all, the classification of the data by stage simplifies the information and therefore decreases its accuracy [34], even though it is needed for analysis and comparison.

Finally, the literature used as reference is old (1994 to 2010) and is derived from different countries (USA, France). However, because of medical progress, better early detection and more comprehensive care during the last years, the delay of cancer diagnosis we found in our study (compared to older study references) has probably been underestimate. New studies should be published using data from the French National Cancer Plan 2009–2013 [18].

We didn’t undertake a socio-economic study of our population. However, we know that deaf people in France have a slightly lower socio-economic level than the general population [35] and that it depends on the socio-professional category. Thus far, there is no correlation between deaf and cancer.

Before this study, in the literature we find works on transmission of information for Deaf with cancer, but this study is the first study studying the diagnostic stage of cancer in Deaf population. Despite of the difference of existing structures between countries, there is always a difficulty of access to the information, screening programs and health education of deaf people [36, 37].

### Implications into practice

The Deaf have difficulty accessing medical information including a communication barrier with the practitioner and a lack of education and interpreters [5, 9, 10]. In addition; difficulties of communications remain between general practitioners and oncologists [38]. Exams such as mammography and Papanicolau examination are not understood as well by the Deaf although they undergo these tests as often as people with hearing [10, 26, 39]. The impact of the lack of medical knowledge during a medical consultation is a real problem, even in the UASS where the language barrier is not as prominent. Most Deaf people have a general practitioner and, additionally, a UASS practitioner but the language barrier remains. It is always possible to prescribe a screening test such as mammography or to perform a rectal examination but explanation of the exam is often short and misunderstood. Moreover, it is always more difficult to have a conversation concerning symptomatology between a Deaf and a hearing person. If the practitioner cannot understand the symptomatology then he/she is unable to recommend the appropriate diagnostic testing. Socioeconomic status must also be considered, as it also affects the survival of the patient [40, 41].

Solutions are available to appropriately disseminate information such as language-adapted educational programs, often on video [39, 42–45], ideally bilingual (video and text) [46]. It has been observed that Deaf people are very interested in sharing the information with their community. The FSL educational video [47] may be an excellent addition to the Public Health Educational Campaign. Even better, it could be adapted to the Deaf culture by Deaf people themselves [48]. Increasing general health knowledge (short-term and long-term) [49] would certainly help to diagnose cancers earlier in the Deaf community. A raising awareness and an education of staff medical can allow a better scattering of the information [50].

Raising awareness among medical practitioners as to the communication problem existing between Deaf and hearing people would also improve communication and the identification of their diagnoses and health needs.

This study targeted the Deaf population suffering from cancer and evaluated the stage at initial diagnosis. To further establish if a delayed diagnosis does exist in the general Deaf Population it would be important to perform a case-witness study in order to compare Deaf patients with a paired population.

## Conclusion

After review of 4263 Deaf patients’ medical records from the UASS in Grenoble, Poitiers, Paris, Marseille and Toulouse, 80 had a cancer diagnosis. We observed a delay of diagnosis regarding cancers with a complex screening process (colo-rectal cancer), non-systematic screening (prostate cancer and melanoma) and, additionally, with information that was not well codified.

Deafness seems to increase disparity in the access to health care, which could be a factor of bad prognosis in cancers. Many factors could explain this delay such as communication problems between a Deaf patient and a medical practitioner, a lack of general health knowledge within the Deaf Community and the difficult access to written language.

These factors can also become obstacles in the access to the Public Health Campaign and to medical information in general. The UASS helps reduce the language barriers, optimize communication and adapt it to consultations of screening, diagnosis and care of patients. Increasing the population served by the UASS and creating sign language medical information campaigns would improve the lack of general health knowledge in the Deaf community. Mass and individual screenings would also help greatly to an early detection of disease.

## Abbreviations

FET: Fisher’s Exact Test
FSL: French Sign Language
ICD-10: International Classification of Diseases (ICD-10)
PSA: Prostate Specific Antigen
UASS: French Deaf Care Units
USA: United States of America
WHO: World Health Organization
